# Mechanism of tau‐mediated microglial NF‐κB inhibitor IκBα degradation in Alzheimer’s disease

**DOI:** 10.1002/alz.095143

**Published:** 2025-01-09

**Authors:** Karthikeyan Tangavelou, Shanya Jiang, Michael Mandell, Kiran Bhaskar

**Affiliations:** ^1^ Department of Molecular Genetics and Microbiology, University of New Mexico Health Sciences Center, Albuquerque, NM USA; ^2^ University of New Mexico, Albuquerque, NM USA

## Abstract

**Background:**

Microglia are the brain resident immune cells that function as immune surveillance and engulf and clear damage‐associated molecular patterns (DAMPs), such as misfolded and oligomeric tau (TO) relevant Alzheimer’s disease (AD) and prevent nuclear factor‐kB (NF‐kB) mediated immune‐activation. IκBα is an endogenous inhibitor of the NF‐kB subunit p50‐p65/c‐Rel protein complex. IkBα’s association is precisely regulated in microglia to prevent excessive NF‐kB activation and neuroinflammation, which is one of the hallmarks of AD.

**Methods:**

Microglia treated with tau oligomers (TO) purified from autopsy of the human AD brain and analyzed its uptake and clearance via autophagy, proteasome, or calpain using specific inhibitors, bafilomycin‐A (BafA1), MG‐132 and PD150606, respectively. TO’s effect on NF‐kB activity (via assessing IkBα levels) was also tested in the presence or absence of these inhibitors and upon inhibition of protein translation using cycloheximide.

**Results:**

AD‐TO induces endomembrane damage and NF‐kB activity as confirmed by increased galectin 3 puncta and luciferase reporter activity, respectively. AD‐TO impairs autophagy, confirmed by accumulation of p62, and adding BafA1 does not increase either p62 or IkBα levels. Then, to determine the role of proteasome or calpains on IkBα degradation, AD‐TO induced IκBα degradation was attempted to stabilize by either MG‐132 or PD150606. Inhibiting either proteasome or calpains does not prevent the degradation of IkBα. We identified AD‐TO induces elevated calpain activity, which is correlating with autophagy impairment, and inhibiting calpain with PD150606 restored autophagy activity as confirmed by decreased intracellular level of p62 and AD‐TO. Inhibiting the proteostasis activity with either PD150606 + BafA1 or PD150606 + BafA1 + MG‐132 induces secretion of IkBα into the culture medium.

**Conclusions:**

Microglial endocytosed AD‐TO damages endomembrane and activate NF‐kB by constitutively degrading IkBα, which localizes in autophagosomes, and concurrent inhibition of calpains and autophagy leading to secretion of IkBα into the extracellular space.

